# Supplementary figures and images for: Intermedilysin cytolytic activity depends on heparan sulfates and membrane composition
Source: PLoS Genet. 2021 Feb 12;17(2):e1009387. doi: 10.1371/journal.pgen.1009387 (PMC7906465; doi:10.1371/journal.pgen.1009387)

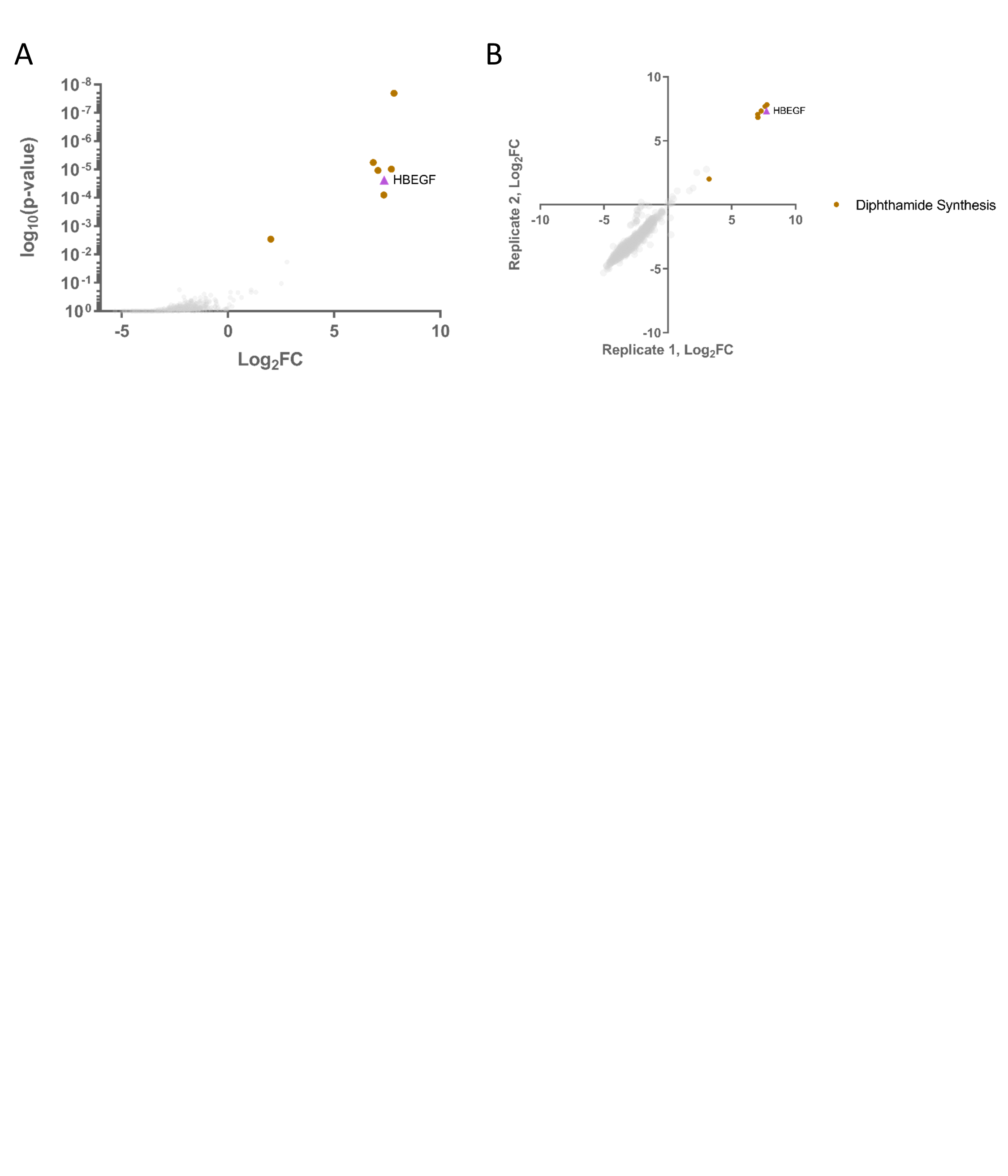

Supplement: S1 Fig — A. Volcano plot of a representative DT genome-wide CRISPR screen replicate. Y-axis displays the significance of the hit, while the X-axis represents Log2-fold change in the average number of guide reads (of all four guides) when compared with non-selected cells. B. Results of two independent DT CRISPR knock-out screens. Axes represent Log2-fold changes in the average number of guide reads (of all four guides) when compared with non-selected cells from two independent replications of the screen. We identified all of the genes involved in the diphthamide biosynthesis and the toxin receptor–HBEGF. (TIF) [file pgen.1009387.s003.TIF]

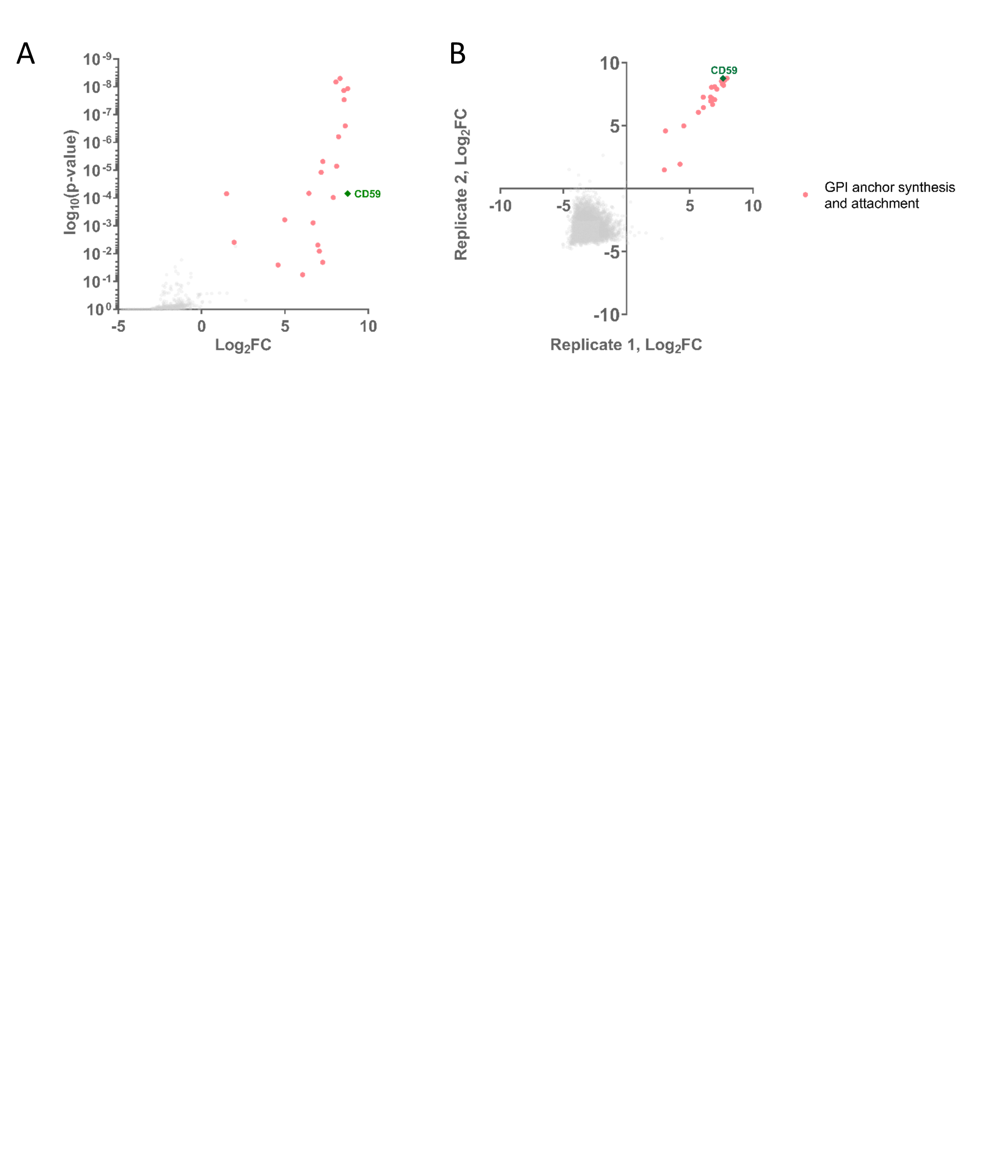

Supplement: S2 Fig — A. Volcano plot of the representative ILY genome-wide CRISPR screen after the second round of selection with 10 ng/ml ILY. Axes represent Log2-fold changes in the number of the average number of guide reads (of all four guides) when compared with non-selected cells from two independent replications of the screen. We identified most of the genes in GPI anchor synthesis and attachment cascades as well as the receptor of ILY–CD59. B. Results of two independent CRISPR knock-out screens after the second round of selection with 10 ng/ml ILY. Axes represent Log2-fold changes in the average number of guide reads (of all four guides) when compared with non-selected cells from two independent replications of the screen. (TIF) [file pgen.1009387.s004.TIF]

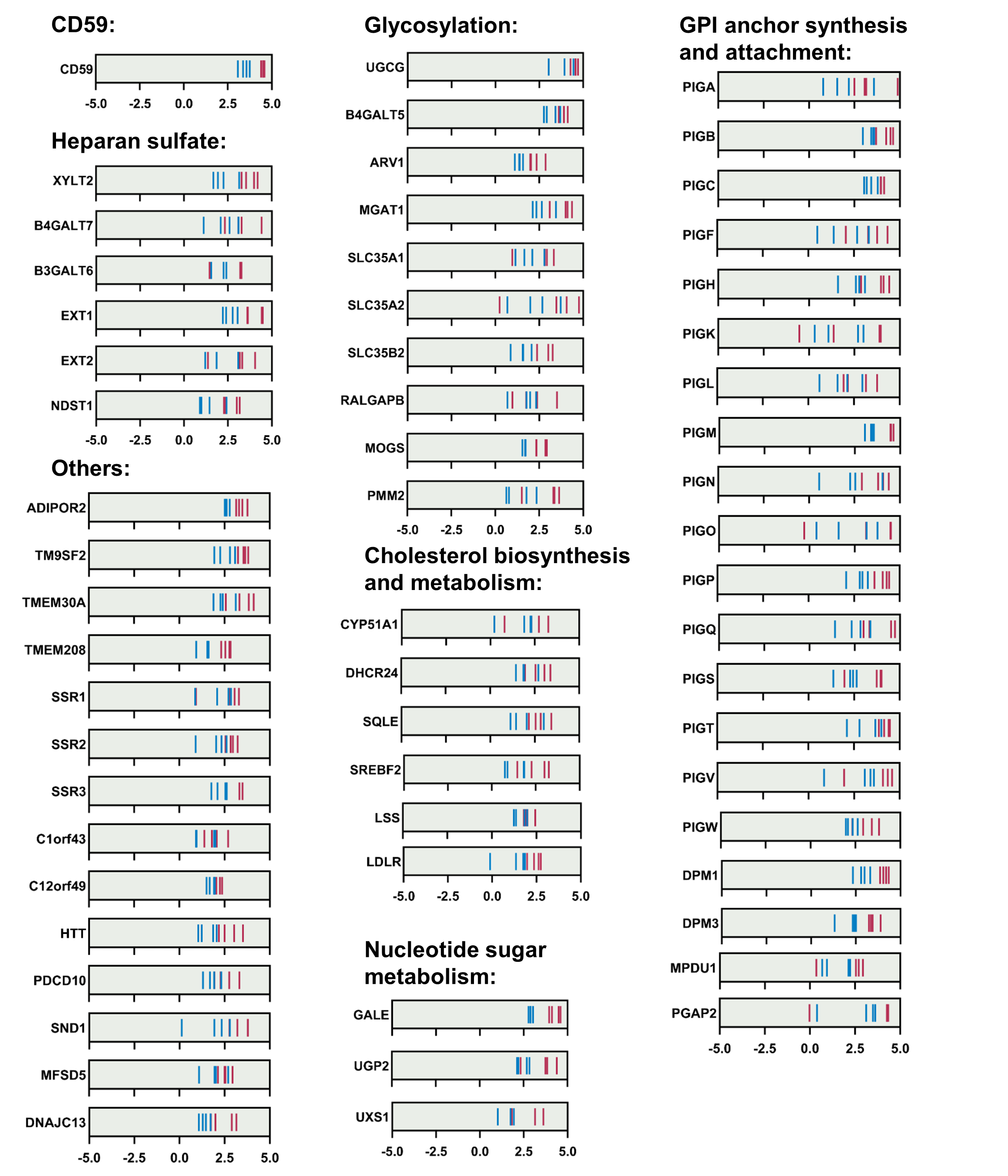

Supplement: S3 Fig — Different colors of dashes represent Log2-fold enrichments of guides from different independent ILY screens—guide RNA enrichment from the first screen depicted in blue, guide RNA enrichment from the second screen depicted in red. (TIF) [file pgen.1009387.s005.TIF]

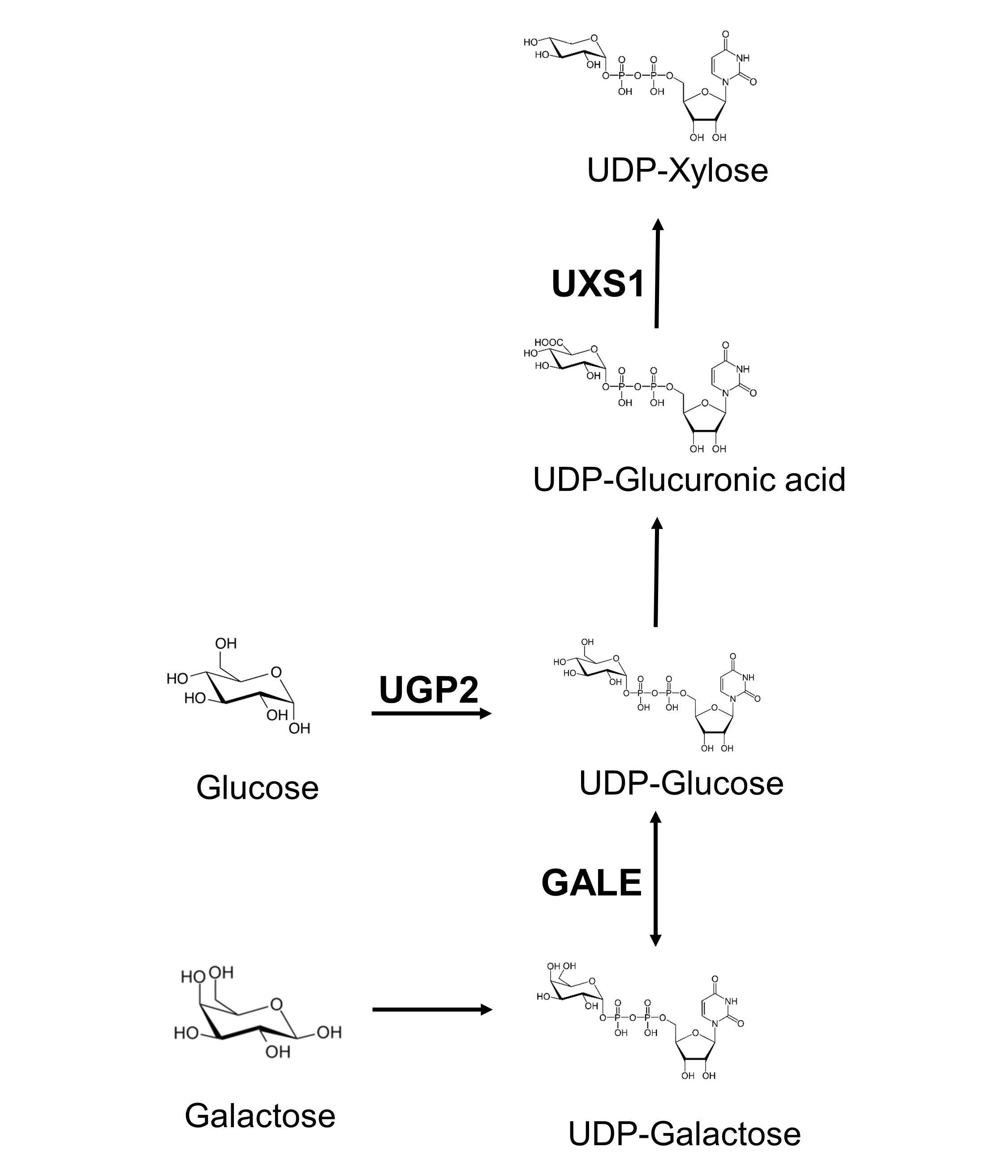

Supplement: S4 Fig — Genes listed on the arrows were identified in this study. (TIF) [file pgen.1009387.s006.TIF]

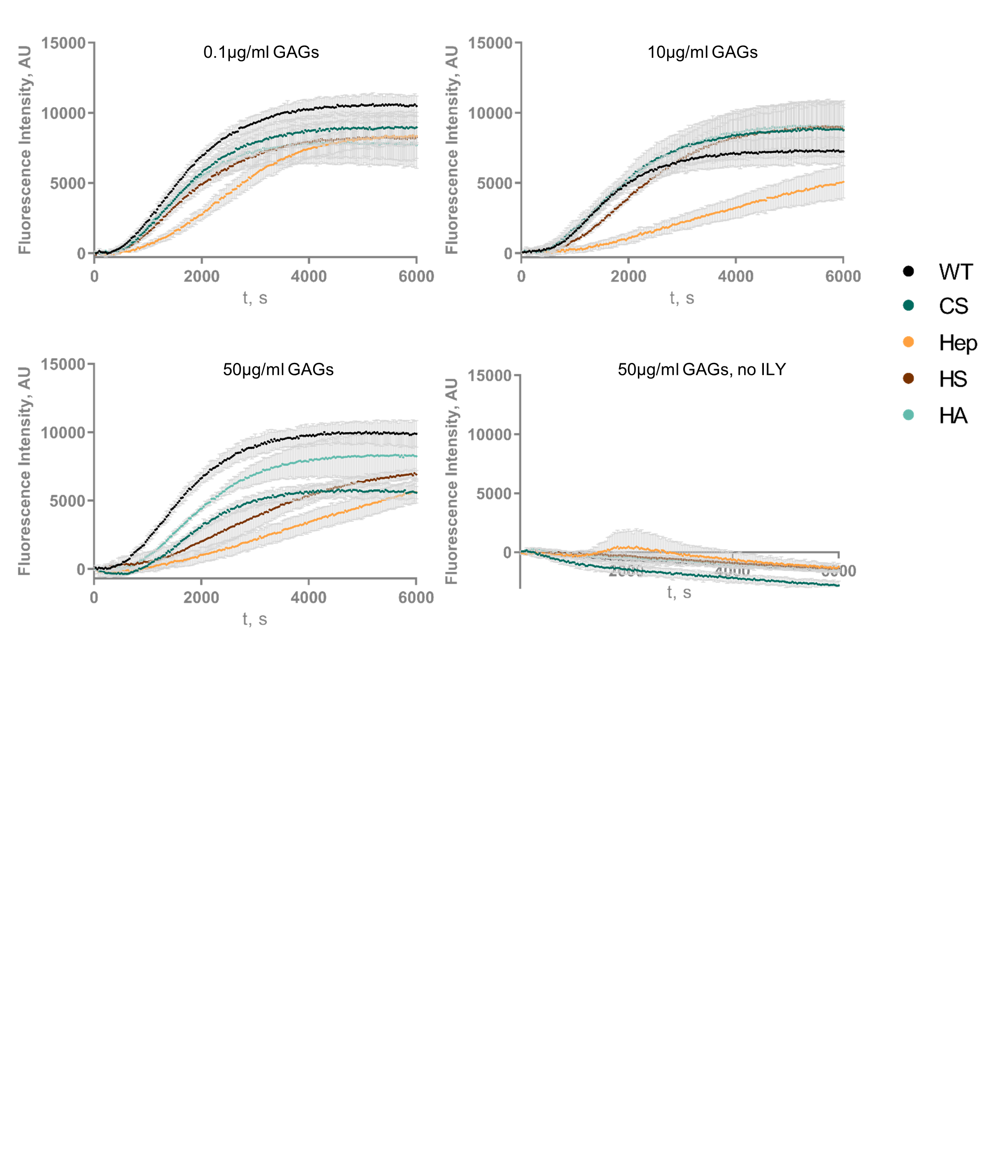

Supplement: S5 Fig — Qualitative kinetics of the ILY pore-formation on the WT cells with added different glycosaminoglycans (GAGs) at various concentrations. WT–no GAG added; CS–chondroitin sulfate; Hep–heparin; HS–heparan sulfate; HA–hyaluronic acid. Higher fluorescence intensity corresponds to more propidium iodide entering pores formed by ILY and is a surrogate measurement of the speed at which pores form and cells are lysed. Heparin and heparan sulfate, at higher concentrations, competitively inhibit ILY. However, at highest concentrations tested other GAGs inhibit ILY as well, suggesting non-specific electrostatic interactions. Bottom left panel represents a control experiment, with no ILY added. n = 3, error bars represent standard deviations. (TIF) [file pgen.1009387.s007.TIF]

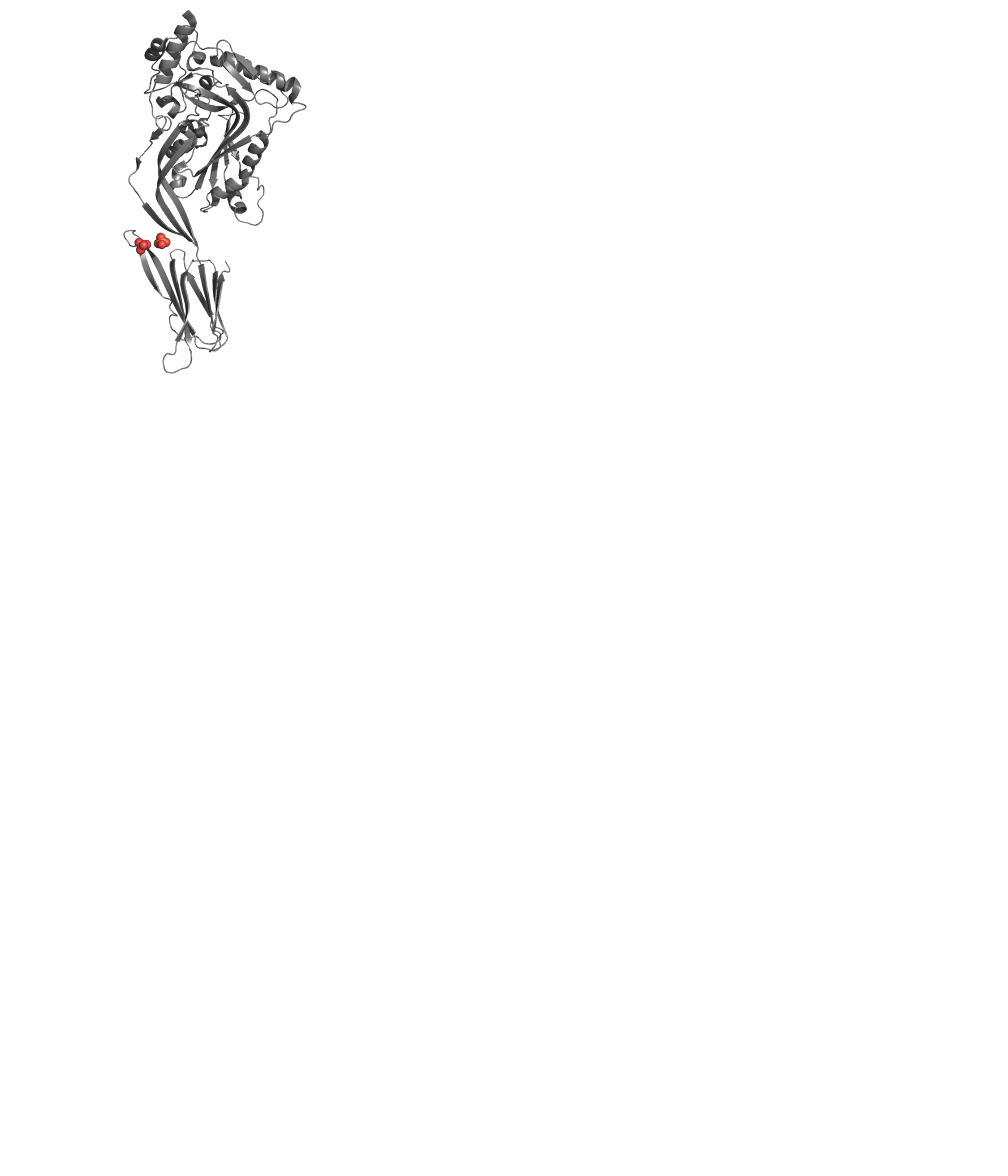

Supplement: S6 Fig — PDB ID: 1S3R. (TIF) [file pgen.1009387.s008.TIF]

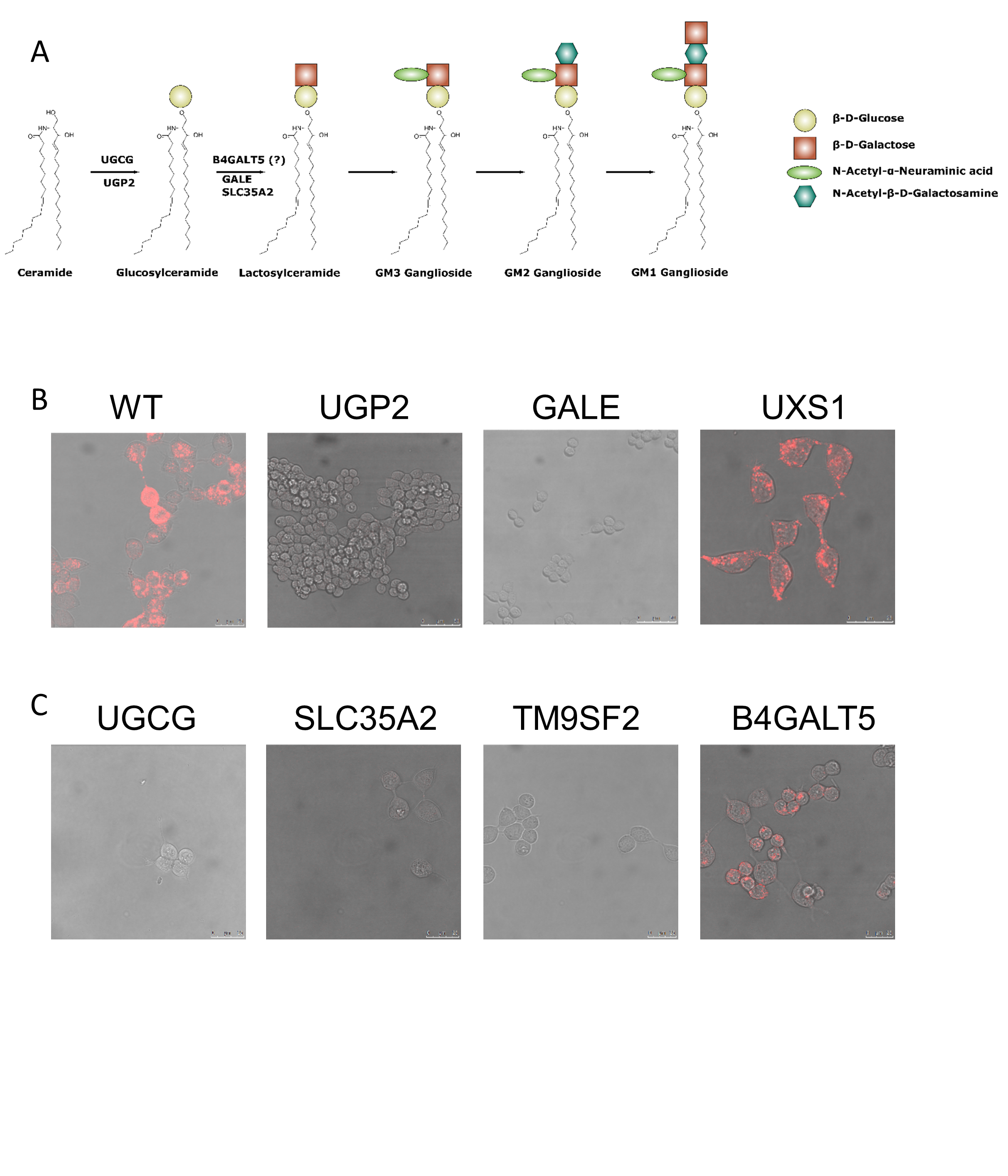

Supplement: S7 Fig — A. Schematic representation of GM1 ganglioside biosynthesis synthesis. Genes listed on the arrows were identified in this study. B. Staining of the cells bearing UDP-sugar gene knock-out with cholera toxin B (CTB) labeled with AlexaFluor594. CTB binds GM1 gangliosides present in the plasma membrane. UGP2 and GALE cells did not stain with the CTB. C. Staining of the cells bearing listed gene knock-out with cholera toxin B (CTB) labeled with AlexaFluor594. CTB binds GM1 gangliosides present in the plasma membrane. UGCG, SLC35A2, and TM9SF2 knock-out cells did not stain with CTB. (TIF) [file pgen.1009387.s009.TIF]

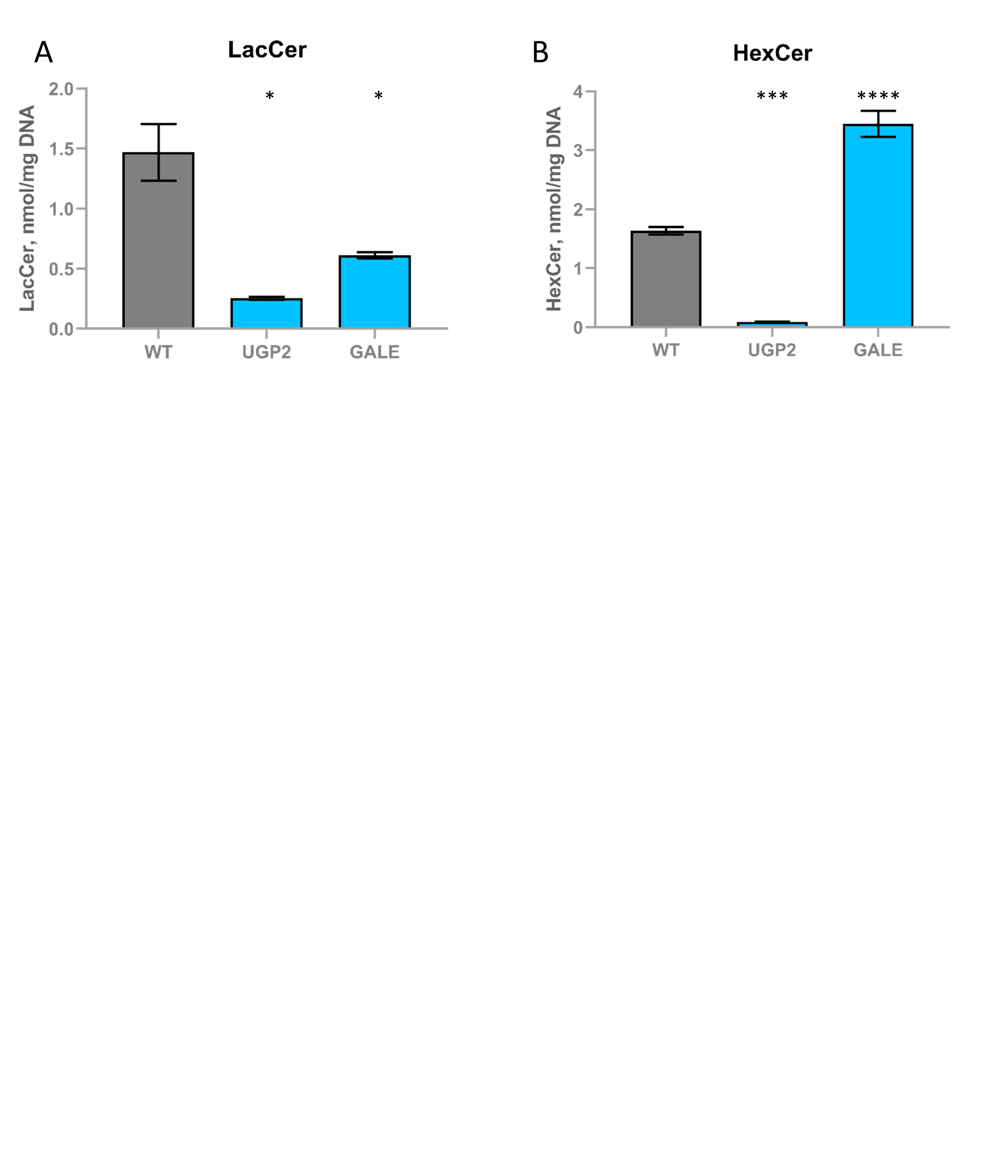

Supplement: S8 Fig — A. Changes in the amount of hexosylceramide present in GALE and UGP2 knock-out cell lines. n = 3, error bars represent standard deviation. p-values were calculated using two-tailed t-test; *–<0.05; **–<0.01, ***–<0.001; ****–<0.0001. B. Changes in the amount of lactosylceramide present in GALE and UGP2 knock-out cell lines. n = 3, error bars represent standard deviation. p-values were calculated using two-tailed t-test; *–<0.05; **–<0.01, ***–<0.001; ****–<0.0001. (TIF) [file pgen.1009387.s010.TIF]

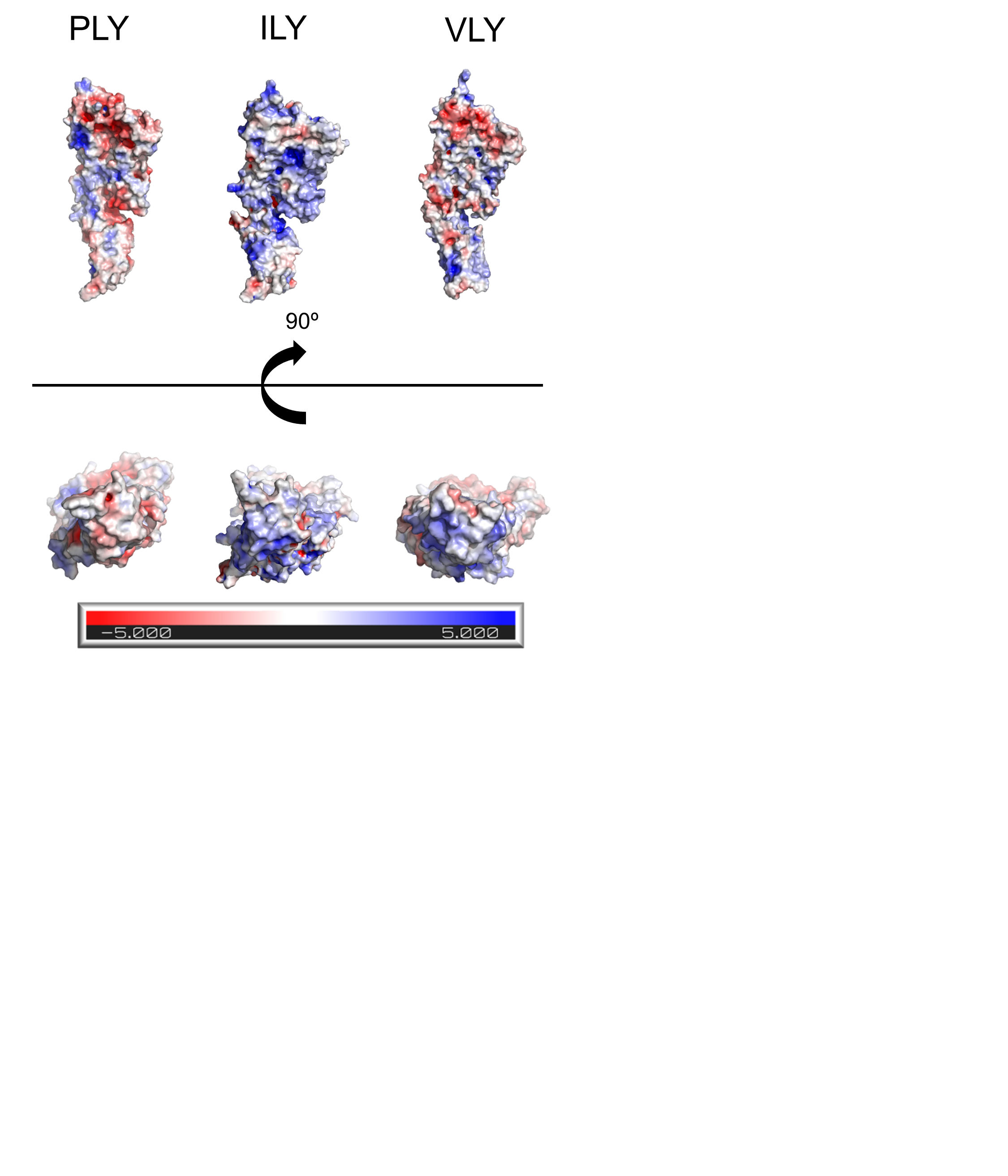

Supplement: S9 Fig — Representation of surface charge distribution of PLY, ILY, and VLY in an overall protein and the bottom, membrane interacting surface. In contrast to VLY and ILY, PLY is more negative overall as well as in the membrane-interacting domain. Representations were generated from the following structures: PDB IDs: 1SR3; 5CR6; 5IMY. Charge distributions were calculated using the Poisson-Boltzmann solver plugin in Pymol. (TIF) [file pgen.1009387.s011.TIF]
