# Supplementary material for: Intermedilysin cytolytic activity depends on heparan sulfates and membrane composition
Source: PLoS Genet. 2021 Feb 12;17(2):e1009387. doi: 10.1371/journal.pgen.1009387 (PMC7906465; doi:10.1371/journal.pgen.1009387)
Supplement: Attachment — Submitted filename: Response letter.docx [file pgen.1009387.s012.docx]

PGENETICS-D-20-01190: point-by-point response to the reviewer’s comments

**Reviewer #1**: *Here the authors carried out genome-wide CRISPR knockout screens for a representative cholesterol-dependent cytolysins (CDC), intermedilysin (ILY). The screen successfully identified the known receptor CD59, and many weak hits. The authors validated many of them and determined that heparan sulfates facilitate the kinetics of forming ILY pore on cell surfaces, likely by mediating toxin attachment onto cells, and disrupting glucosylceramide pathway could also reduce ILY activity. Finally, they examined two other related CDCs and found that one (pneumolysin) showed reduced activity when TMEM30A, a phospholipid flippase is disrupted. These findings are novel and interesting, adding new insight to our understanding of CDCs. The writing and figure organization however need to be further improved.*

We thank the reviewer for the constructive comments and suggestions to improve the manuscript.

*Major issues:*

*The specificity of heparan sulfate for ILY can be further explored to determine whether*

*it is the sulfate group that is responsible for ILY attachment, for instance, by examining competition with other sulfated GAGs and non-sulfated GAG in Fig. 3G.*

This is an excellent suggestion, and accordingly, we have performed competition assays with other differentially sulfonylated GAGs, namely heparan sulfate, chondroitin sulfate, and hyaluronic acid. Heparan sulfate is structurally similar to heparin but less sulfonylated and contains O- and N-sulfonylation; chondroitin sulfate contains only O-sulfate groups, and hyaluronic acid is a non-sulfonylated GAG. We found that heparin has the highest affinity, but heparan sulfate also competitively inhibited ILY. Chondroitin sulfate and hyaluronic acid did trigger a measurable reduction in the pore-formation kinetics. This suggests that N-sulfate, common between heparin and heparan sulfate, is, at least in part, responsible for the observed competitive inhibition. We cannot exclude, however, that other structural or functional elements of these molecules are involved.

The results are presented in Figure 4F and Supplementary Figure 5 and described in an expanded chapter on heparan sulfates in the revised manuscript.

*Minor issues:*

*Line 84: One of the early successful CRISPR-Cas9 screen on bacterial toxin is PMID: 27680706 (Tao et al.).*

This reference is now included in the revised version of the manuscript

*Line 98: use ILY, no need to use intermedilysin after the first time.*

This is adapted throughout the text except in the chapter subheadings.

*Line 104: there should be a space between number and unit. Please check.*

This is now corrected.

*Line 113: Diphtheria toxin is more commonly called DT, not DFT.*

This is now corrected.

*Line 227 and Suppl. Fig. 7, UGCG is required not just for gangliosides, but also other glycolipids such as Gb3. It was also a hit in recent Shiga toxin screens.*

This is now mentioned in lines 252 and 253 of the revised manuscript.

*Line 635: The title needs to be changed. E.g. Results of two independent ILY CRISPR KO screens.*

This is now changed in the revised manuscript to: “Intermedilysin genome-wide CRISPR knock-out screening identifies novel genes involved in intermedilysin-mediated cell lysis”

*HS pathway is frequently identified in screens for many pathogens, viruses, and also for bacterial toxins (e.g. PMID: 31160825). The authors can add a short discussion on it.*

We have now added a brief discussion at the end of the discussion section on the potential role of heparin in the body. Lines 240 and 241.

*Fig. 1D: is the unit ng/ml? there is no need to go up to 150 on y-axis.*

That is correct. This is now fixed in this and subsequent figures of the revised manuscript.

*Fig. 2A, 3A: It would be better to remove these panels, and directly note the hits on an enlarged Fig. 1C.*

We thank the reviewer for the suggestion, and we removed 2A, 3A, and Supplementary figure 5 and instead labeled all of the hits in the expanded figure 1B.

*Fig. 3E: Is the XYLT2 the same data as in panel B? No need to replot it here.*

We agree and decided that figure 3E did not add to the data and thus removed it.

*Fig. 4C: this panel can be moved to supplementary figures, as evidence for membrane charge contribution is weak.*

*Move supplementary Fig. 6 and 13 into main figures – these data represent lots of work and are interesting.*

We have done as suggested in the revised manuscript.

**Reviewer #2**: *The current study describes a genome-wide Crispr/Cas9 screen of cellular factors responsible for intermedilysin membrane binding/cell lysis.
I quite liked this paper – the study is well-done, interesting, logical and well-written. The authors have identified a number of novel genes responsible for ILY lysis, and the targets have been carefully validated. As with any other screen, the current paper raises many potentially interesting questions for future studies.*

We thank the reviewer for the constructive comments and suggestions to improve the manuscript.

*There two points that I feel the authors should address.*

1. *Since CD59 is a plasma membrane glycoprotein, it should be demonstrated that none of the positive hits alter its surface levels – altered glycosylation may influence the stability of CD59 and lipid redistribution may influence its surface exposure. What is the glycosylation status of CD59, ie does it contain heparan sulfate moiety? If so, does it regulate ILY-CD59 interaction? This can be potentially validated by mutating the O-linked glycan sites and re-expressing the mutant protein in CD59-knockout cells.*

We thank the reviewer for this great suggestion! We performed the suggested experiment and measured CD59 surface levels using FACS and found that N-glycosylation defects indeed caused a reduction in surface CD59. We included the data in the new Figure 2B and included a description of the results in the new section “Defects in N-glycosylation and ER translocation reduce plasma membrane levels of CD59”. However, at the moment, we cannot definitively confirm whether this effect is caused by global defects in N-glycosylation or by CD59 specific N-glycosylation defect. This is also mentioned as an open question in the new chapter.

To the best of our knowledge and according to mass spectrometry studies of glycosylation profiles of CD59 (e.g., Rudd *et al*., 1997; PMID: 9054419), CD59 does not contain heparan sulfate moiety.

2. *How do the authors distinguish ILY membrane binding and failure to lyse the cells, from the lack of membrane binding? This can be assessed by the Western immunoblotting of membrane-bound ILY, if there are antibodies available or the protein is tagged for purification (it is unclear how ILY was purified), or by flow cytometry. The reason for this question is a recent study by Rudd-Schmidt et al (2019) where another pore-forming protein, perforin, was shown to bind to PS but could not lyse the cells.*

We have now included a brief description of how the toxins were prepared in the methods section. The purification tag had been cleaved off.

This is a great observation, and we indeed do not know whether ILY is unable to bind to the membrane or is unable to perforate it in our ILY-resistant cell lines. Although we agree that this is an interesting question, it is somewhat beyond the scope of the present study, and answering it definitively would require an elaborate investigation. The reason why is that CD59 is the primary receptor initiating ILY binding to the membrane, and it has been demonstrated that ILY cannot bind to cells lacking CD59 (e.g., Giddings et al., 2004; PMID: 15543155). Upon binding to CD59, ILY oligomerizes to form a prepore, which then releases CD59 and transects the membrane (Boyd et al., 2016, PMID: 27910935). Therefore, the amount of total ILY level on the membrane would represent a cumulative effect of both ILY binding CD59 and ILY oligomerization, making it difficult to interpret the contributions of any genetic perturbations that do not eliminate ILY binding. It would be possible to eliminate ILY binding to the cells due to oligomerization using mutant ILY proteins incapable of oligomerization; however, they would be incapable of lyzing the cells, thus removing us further from the aim of the study.
