# Supplementary material for: Intermedilysin cytolytic activity depends on heparan sulfates and membrane composition
Source: PLoS Genet. 2021 Feb 12;17(2):e1009387. doi: 10.1371/journal.pgen.1009387 (PMC7906465; doi:10.1371/journal.pgen.1009387)
Supplement: Attachment — Submitted filename: Response letter.docx [file pgen.1009387.s013.docx]

PGENETICS-D-20-01190R1: point-by-point response to the reviewer’s comments

***Reviewer #1****: The authors have done a great job revising the manuscript and I only have a few minor edits:*

We thank the reviewer for the suggestions to further improve the manuscript.

*Line 28: Cas9.*

This is now corrected.

*Line 36: delete “additionally”.*

This is now deleted.

*Line 41: delete “however”.*

This is now deleted.

*Line 89: delete “however”.*

This is now deleted.

*Line 252: delete “recently identified as” and change it to “which is the receptor for Shiga toxin”.*

This is now corrected as the reviewer suggested.

*Line 761: change the title for this figure legend to directly mention VLY and PLY.*

The figure legend now directly mentions VLY and PLY.

**Reviewer #2**:  I appreciate the authors' effort in answering my queries.
I have noticed though that there was no clear correlation between the effect of gene knockout on CD59 expression and cytolysis. The authors rightly pointed out that they could not distinguish the effect of altered N-glycosylation on CD59 surface expression from other (eg global) effects of gene knockouts.

I apologise for being a pain, but they can in fact resolve that conundrum, or at least make a step in that direction, by conducting a simple experiment. They should knock-out CD59 using crispr, and then re-express it (eg by viral transduction). What they'll have as a result is cells that produce a wide range of CD59 protein. Then they can then sort various populations of cells that would match their other cell lines in terms of CD59 expression levels. In addition, the authors can treat the cells with ILY and assess CD59 expression levels before and after adding ILY - that way they should see an enrichment of CD59 low/negative population etc.

The outcome of these experiments is that they will be able to determine the minimum level of CD59 that will affect ILY lytic activity and, potentially, uncouple the effect of their glycosylation genes on CD59 from other effects that they might cause. It is possible, for example, that the diminished activity of ILY with respect to UGP2-knockout cells has nothing to do with the extent of the reduction of CD59 expression, or they might find an excellent correlation.

We thank the reviewer for the constructive comments to improve the manuscript. This is indeed a good experiment to discriminate the effect of CD59 glycosylation from the other effects. However, although it appears a simple experiment, it is quite elaborate and time-consuming to create suitable stable cell lines with the various expression levels and correctly characterize them. It can take several months to create stable cell lines. Additionally, to draw a correct conclusion, we will need to create CD59 glycosylation mutants as controls, which may introduce additional variables due to the introduction of mutations into the native CD59 protein.

Moreover, our study is mainly about discovering novel factors in genome-wide screening. We agree that it is interesting to know in greater detail how these novel factors influence ILY, and thanks to the previous comment of the reviewer, we were able already to provide a strong basis for the mechanism of action studies. We believe that the effect we now observe on CD59 expression levels with a few novel genes (Figure 2B) already provides a good direction for a follow-up study to elaborate on our findings here. While we are aware of the questions the reviewer raises and acknowledge their importance, we feel it is beyond the scope of the present study and deserves a separate, more detailed work to address. We hope the reviewer understands our arguments, and we hope the reviewer still finds this study in its current form suitable for publication.
